# Biodegradation of Unpretreated Low-Density Polyethylene (LDPE) by *Stenotrophomonas* sp. and *Achromobacter* sp., Isolated From Waste Dumpsite and Drilling Fluid

**DOI:** 10.3389/fmicb.2020.603210

**Published:** 2020-12-16

**Authors:** Anindya Sundar Dey, Himadri Bose, Balaram Mohapatra, Pinaki Sar

**Affiliations:** Department of Biotechnology, Indian Institute of Technology Kharagpur, Kharagpur,, India

**Keywords:** LDPE, biodegradation, *Stenotrophomonas*, *Achromobacter*, bioremediation

## Abstract

Exploring the catabolic repertoire of natural bacteria for biodegradation of plastics is one of the priority areas of biotechnology research. Low Density Polyethylene (LDPE) is recalcitrant and poses serious threats to our environment. The present study explored the LDPE biodegradation potential of aerobic bacteria enriched from municipal waste dumpsite and bentonite based drilling fluids from a deep subsurface drilling operation. Considerable bacterial growth coupled with significant weight loss of the LDPE beads (∼8%), change in pH to acidic condition and biofilm cell growth around the beads (CFU count 10^5^–10^6^/cm^2^) were noted for two samples (P and DF2). The enriched microbial consortia thus obtained displayed high (65–90%) cell surface hydrophobicity, confirming their potential toward LDPE adhesion as well as biofilm formation. Two LDPE degrading bacterial strains affiliated to *Stenotrophomonas* sp. and *Achromobacter* sp. were isolated as pure culture from P and DF2 enrichments. 16S rRNA gene sequences of these isolates indicated their taxonomic novelty. Further biodegradation studies provided strong evidence toward the LDPE metabolizing ability of these two organisms. Atomic Fore Microscopy (AFM) and Scanning Electron Microscopy (SEM) revealed considerable damage (in terms of formation of cracks, grooves, etc.) on the micrometric surface of the LDPE film. Analysis of the average roughness (Ra), root mean square roughness (Rq), average height (Rz), maximum peak height (Rp), and maximum valley depth (Rv) (nano-roughness parameters) through AFM indicated 2–3 fold increase in nano-roughness of the LDPE film. FTIR analysis suggested incorporation of alkoxy (1000–1090 cm^–1^), acyl (1220 cm^–1^), nitro (1500–1600 cm^–1^), carbonyl (1720 cm^–1^) groups into the carbon backbone, formation of N-O stretching (1360 cm^–1^) and chain scission (905 cm^–1^) in the microbially treated LDPEs. Increase in carbonyl index (15–20 fold), double bond index (1.5–2 fold) and terminal double bond index (30–40 fold) confirmed that biodegraded LDPEs had undergone oxidation, vinylene formation and chain scission. The data suggested that oxidation and dehydrogenation could be the key steps allowing formation of low molecular weight products suitable for their further mineralization by the test bacteria. The study highlighted LDPE degrading ability of natural bacteria and provided the opportunity for their development in plastic remediation process.

## Introduction

Despite being a severe threat to the environment, plastic has become a very fundamental part of human society (Danso et al., 2019). It is widely used in various fields ranging from industries, agriculture to our day to day life, due to its lightness, durability, inertness and cheapness, etc., (Orr et al., 2004; Sudhakar et al., 2008; Yuan et al., 2020). Plastics are polymers of small aliphatic (for example polyethylene is made of [CH_2_–CH_2_] monomer) or aromatic molecules (e.g., polystyrene is a polymer of styrene) and their derivatives (such as polyvinyl chloride made from vinyl chloride monomer) (Andrady and Neal, 2009; Yang et al., 2014). Almost 6.3 billion tons of plastics were produced worldwide in 2015 and the number is increasing exponentially each year because of its efficient and versatile use (Yuan et al., 2020). Although plastics bring ease to our daily life, their uncontrolled use and careless disposal have been imposing a constant threat to the ecosystem, since they don’t get degraded naturally even after many years and interfere with various natural and engineered processes adversely (Albertsson and Karlsson, 1990; Tokiwa et al., 2009; Raddadi and Fava, 2019). Non/slow biodegradation of plastics has led to their accumulation in the environment, thus causing wide spread pollution and harming marine as well as terrestrial life forms (Raddadi and Fava, 2019; Li et al., 2020). Plastics not only cause flooding by blocking the water draining system, but also get incorporated into the food chain of animals and damage their digestive system (Teuten et al., 2009; Muhonja et al., 2018). Long term accumulation of plastics in soil even changes its microbial community structure (Harshvardhan and Jha, 2013; Huang et al., 2019; Fei et al., 2020). Although, the amount of plastic wastes is reduced by incineration, it leads to secondary pollution due to the production of air pollutants like carbon monoxide, nitrogen oxides, etc., (Ru et al., 2020). The weathering and breaking down of plastic produce microplastics (MP) which migrate toward rivers, ponds, lakes, oceans and agricultural fields and affect them adversely (Yuan et al., 2020). Most of the commercially used plastics like polyethylene (PE) (low density, i.e., LDPE and high density or HDPE), polypropylene, polystyrene, polyvinyl chloride (PVC), polyamide (PA), polyethylene terephthalate (PET) are usually resistant toward biodegradation (Tokiwa et al., 2009; Danso et al., 2019). Their biodegradability is generally hindered due to several factors such as, (1) not being able to enter the microbial cell because of high molecular weight, (2) better stability in chemical structure, (3) absence of functional groups where microbial enzymes can attack and (4) high hydrophobicity and degree of crystallinity due to large carbon backbone (Tokiwa et al., 2009; Yang et al., 2014). Environmental toxicity, large-scale accumulation and persistence of plastics warrant immediate action on development of efficient and ecofriendly methods for their degradation and exploration of microbial catabolic potential toward biodegradation of plastics (Montazer et al., 2018; Danso et al., 2019).

It has been found that the most common plastic, e.g., polyethylene (PE) can be subjected to microbial degradation if it is made with added pro-oxidants (generally transition metals) (Lee et al., 1991; Bonhomme et al., 2003). It has been observed that microbial growth and thus degradation of PE can be facilitated by several pretreatments like photo-oxidation (by exposing under UV irradiation), heat treatment (heating with high temperature) or chemical treatment (using acid). These pretreatments usually reduce the hydrophobicity by incorporating various functional groups (carbonyl, keto, nitro, etc.) into the inert carbon backbone (Albertsson et al., 1987; Orr et al., 2004; Hadad et al., 2005). The complex process of complete biodegradation of polyethylene follows these steps: (a) fragmentation by microbial intervention (adhering to the surface of polyethylene) or environmental components and incorporation of functional groups if applicable; (b) breaking down of polymer into oligomers or monomers as well as fatty acids, ketons, aldehydes, alcohols, etc., by enzymatic attack and free radicals; (c) uptake of these small products inside the microbial cells; (d) utilization of those molecules in cellular metabolism and finally production of CO_2_, N_2_, CH_4_, H_2_O, etc., (Yang et al., 2014; Wilkes and Aristilde, 2017; Muhonja et al., 2018; Delacuvellerie et al., 2019; Park and Kim, 2019). Both culture-based and culture-independent metagenomic studies have highlighted the PE (LDPE or HDPE) biodegradation abilities of several bacterial taxa *viz*. *Enterobacter, Bacillus* (Yang et al., 2014); *Brevibacillus* (Hadad et al., 2005); *Pseudomonas* (Muhonja et al., 2018); *Alcanivorax, Ideonella, Marinobacter, Arenibacter* (Delacuvellerie et al., 2019)*; Aneurinibacillus* (Raddadi and Fava, 2019)*; Chelatococcus* (Yoon et al., 2012); *Achromobacter* (Kowalczyk et al., 2016); *Comamonas, Stenotrophomonas* and *Delftia* (Peixoto et al., 2017). Besides, several members of soil-inhabiting *Actinobacteria* (*Rhodococcus* sp., *Streptomyces coelicoflavus*, *Streptomyces* KU1, KU5, KU6, KU8, *Streptomyces werraensis*, *Streptomyces humidus*, *Streptomyces parvullus*, *Streptomyces aburaviensis*, *Amycolatopsis* sp. HT-32, *Nocardia* sp. *Saccharothrix wayandensis*, *etc*.) have shown either weight reduction or partial degradation of PE films (Sivan et al., 2006; Abraham et al., 2017; Huang et al., 2019; Soulenthone et al., 2020). Among the fungal species, members of *Aspergillus* (Muhonja et al., 2018); *Fusarium* (Sivan, 2011); *Penicillium, Zalerion* (Raddadi and Fava, 2019); *Chaetomium* and *Pullularia* (Sudhakar et al., 2008) are well known for biodegradation of LDPEs and HDPEs. Microbial enzymes playing important role in biodegradation of PE are identified to be proteases, lipases, cutinases, laccases, manganese peroxidases, lignin peroxidases, alkane hydroxylases, etc., (Tokiwa et al., 2009; Bhardwaj et al., 2013; Wei and Zimmermann, 2017; Ahmed et al., 2018). Most of the prior studies have been done to assess the biodegradation of pretreated PEs. It is also observed that LDPE biodegradation takes place in a very slow manner even after the pretreatment (Sivan, 2011). In comparison to these studies, biodegradation of un-pretreated LDPE is not yet well studied. Identification and characterization of microbial enzymes and their molecular mechanisms have been considered to be critical in order to develop biotechnological process for plastic remediation (Danso et al., 2019).

The present study was conducted as a first step toward development of microbial plastic bioremediation through evaluating the potential of natural microbial communities and isolating suitable bacterial strains with desirable biocatalytic abilities. LDPE biodegradation potential of naturally occurring aerobic microorganisms (present in municipal waste dump site soil and bentonite based drilling fluids used during deep drilling of igneous crust of Deccan Traps, India) was evaluated by using enrichment culture technique. Degradation of LDPE by the enriched microorganisms was evaluated through a number of analytical techniques including examining the weight loss (of LDPE), change in its surface morphology (SEM and AFM) and chemical modification (FTIR). Two selected bacterial strains isolated from these enrichments were further studied for their individual ability of LDPE biodegradation. These strains were identified through 16S rRNA gene sequencing.

## Materials and Methods

### PE Materials, Samples for Microbiological Isolation, Media and Growth Conditions

Two low-density polyethylene (PE) materials were used in this study as the carbon source: (a) Low Density Polyethylene beads (LDPE beads) (Sigma-Aldrich, United States) (b) Low Density Polyethylene plastic films (LDPE plastic films) (Ranco Poly Bags, Haryana, India). Before use, LDPE beads and films were surface sterilized by washing with 70% ethanol and followed by exposing them to UV rays for 15 min.

Two types of environmental samples were used as the source of LDPE degrading microorganisms: (1) waste plastic sample from landfill soil and (2) drilling fluid. Waste plastic samples were collected from the local landfill site (designated as P) inside the IIT Kharagpur campus (22° 18’ 45.3” N 87° 19’ 28.1” E). The drilling fluid (designated as DF2, DF4, DF7) samples were collected during a 3 km deep pilot borehole (KFD-1) drilling in the Koyna region (17° 24’ 6” N 73°45’8” E) of the Deccan Traps, Maharashtra under the pilot phase of the Koyna scientific drilling project (Bose et al., 2020; Roy and Bansal, 2020). The various depths were as follows: DF2: 1901.255 mbs; DF4:2335.62 mbs; DF7: 2908.52 mbs (mbs: meters below surface). Samples were collected in sterile autoclaved bags with the help of sterile equipment. The DF samples were kept at 4 °C during transportation to lab and thereafter they were transferred to −80 °C and stored until further processing. Dumpsite plastic samples were collected from five sites and stored at 4 °C. They were mixed to use as an inoculum for setting the enrichment within 48 h of sample collection. Drilling fluid (1 g each) samples were incubated with 10 mL sodium pyrophosphate (0.1% w/v) overnight under shaking condition to dislodge the cells, and one mL of the supernatant was used as final inoculum.

The carbon free basal medium used for the enrichment of LDPE degrading bacteria had the following composition: 12.5 g/L K_2_HPO_4_; 3.8 g/L KH_2_PO_4_; 1.0 g/L (NH_4_)_2_SO_4_; 0.1 g/L MgSO_4_⋅7H_2_O (pH 7.0) and 5 mL trace element solution was mixed in 1 L of the medium. The trace element solution consisted of the following: 0.232 g/L H_3_BO_3_; 0.174 g/L ZnSO_4_⋅7H_2_O; 0.116 g/L FeSO_4_(NH_4_)_2_SO_4_⋅6H_2_O; 0.096 g/L CoSO_4_⋅7H_2_O; 0.022 g/L (NH_4_)_6_Mo_7_O_24_⋅4H_2_O; 8.0 g/L CuSO_4_⋅5H_2_O; 8.0 g/L MnSO_4_⋅4H_2_O (Kyaw et al., 2012). Reasoner’s 2A agar (modified to reduce the carbon content by 10 fold) was used for isolation of bacterial strains containing the following components: 0.05 g/L Yeast extract, 0.05 g/L Peptone, 0.5 g/L Casamino acid, 0.05 g/L Glucose, 0.05 g/L Starch, 0.3 g/L K_2_HPO_4_, 0.05 g/L MgSO_4_, 0.3 g/L Sodium Pyruvate; 15 g/L Agar. The composition of modified Luria Bertani (LB) agar medium (M/5) used to determine the biofilm cell growth was as follows: Casein enzyme hydrolyzate 2 g/L, Yeast extract 1 g/L, Sodium chloride 2 g/L, Agar 15 g/L (pH = 7.5 ± 0.2). All the enrichment setups were incubated aerobically at 30 °C in an incubator shaker at 150 rpm for 100 days.

### Enrichment (Setup) and Isolation of PE Degrading Bacteria

P (approximately 10 pieces) and DF (DF2, DF4, DF7) (1 mL) samples were inoculated in 100 mL of basal medium with LDPE as C source. Ten sterilized LDPE beads of around 0.3 g were used as the sole carbon source. Along with that, separate negative control (without inoculum) was prepared. The samples along with negative control (without bacterial inoculum) were kept at 30 °C for 100 days in an incubator shaker (150 rpm). All experiments were set up in duplicates.

After incubation, biofilm or the bacteria present on the surface of the plastic beads were collected according to Kyaw et al. (2012). The beads were immersed in 2 mL of 0.9% NaCl. They were incubated overnight at room temperature. After incubation, they were vortexed properly for few minutes in order to prepare a cell suspension. Hundred μL liquid cell suspension from each sample was inoculated on the modified R2A agar and kept for incubation at 30 °C for 48 h. The colonies obtained were sub-cultured on the same medium for further studies.

### Measurement of Microbial Cell Growth and Change in Medium pH

Following microbial growth, biofilm formation on the surface of the LDPE beads was determined according to the method described by Manijeh et al. (2008). LDPE beads from enrichment cultures were suspended into 0.9% NaCl and vortexed after overnight incubation as described earlier. The resulting suspension was serially diluted within 0.9% NaCl up to 10^5^ fold and 0.1 mL of the diluted sample was plated on modified Luria Bertani Agar plates. The CFU was counted after overnight incubation at 30 °C and the following formula was used to determine the biofilm cell growth.

Biofilm cell growth = log [(average CFU/drop volume) × (dilution counted) × (volume scraped into/surface area)].

In order to measure the microbial growth after incubation, 1 mL of liquid suspension was taken and absorbance (OD_600_) was determined using a UV-VIS spectrophotometer. Similarly, change in pH was also monitored using a pH meter.

### PE Weight Reduction and Hydrophobicity

LDPE beads recovered from the enrichment cultures after 100 days of incubation were washed properly to remove the microbial cells to get the accurate measurement of the weight. These beads were incubated with 2% (w/v) aqueous sodium dodecyl sulfate solution for 4 h in shaking condition, followed by washing with distilled water and 70% ethanol in order to remove the bacterial cells and other cell debris (Orr et al., 2004). The beads were dried at 70 °C. The weight reduction percentage was calculated using the formula mentioned below (Kyaw et al., 2012).

Percentage of Weight Reduction = (Initial Weight – Final Weight) × 100/Initial Weight

Bacterial adhesion to hydrocarbon (BATH) test was carried out to calculate the bacterial cell surface hydrophobicity (Rosenberg et al., 1980). Microorganisms enriched in the different setups were re-inoculated in R2A agar medium and were allowed to grow until they reached mid-logarithmic phase. The microbial cell pellets were obtained by centrifugation and washed with PBS buffer (pH 7.1). The washed cell pellets were re-suspended in fresh PBS buffer. Aliquots of 4 mL each were transferred from the resuspension to fresh test tubes and 1.5 mL of hexadecane was added to them. Solution was vortexed for 2 min and kept still for 15 min to obtain the phase separation. The optical density of the separated phase was measured at 600 nm (OD_600_). Cell-free buffer served as blank. Adherence percentage to hexadecane or BATH was determined using the following formula (Tribedi and Sil, 2013).

Cell surface hydrophobicity (%) = 100 × {(initial OD – final OD)/initial OD}

### Isolation and Identification of LDPE Degrading Bacterial Strains

Following 100 days of incubation, microbial cells present on the LDPE surface of the enrichments, which displayed better plastic degradation, were dislodged in 0.9% NaCl. Hundred μL of cell suspension was plated on modified R2A agar supplemented medium and incubated for 48 h to obtain morphologically distinct colonies. The obtained isolates were routinely sub-cultured on same medium at 30 °C and preserved with glycerol (25%, v/v) at −80 °C. The genomic DNA of isolates (P2, DF22) was extracted using DNA minikit protocol following SDS-Lysozyme lysis and subsequent purification using phenol:chloroform:isoamyl alcohol and ethanol (Mohapatra et al., 2018). The 16S rRNA gene was amplified by PCR with bacterial universal primers (27F: 5′-AGAGTTTGATCCTGGCTCAG-3′ and 1492R: 5′-TACCTTGTTACGACTT-3′). The PCR cycle composed of an initial denaturation step at 95 °C for 5 min, 35 cycles of 95 °C for 45 s, 58 °C for 45 s, 72 °C for 1.5 min, and with a final extension of 72 °C for 7 min. The PCR products were gel purified using a Qia-quick gel extraction kit (QIAGEN), cloned into pTZ57R/T vector (InsTA clone kit, Thermo Fisher Scientific, Waltham, MA, United States), and sequenced using 27F and 1492R, followed by extraction of true nucleotide positions using BioEdit version 7.2.5. Assembly of the whole stretch was done using CAP contig assembly program of BioEdit to obtain ∼1000 bp sequence of 16S rRNA gene. Homology search for maximum similarity of the 16S rRNA gene sequences was carried out using identity tool of EzTaxon-e server^1^ and BlastN of NCBI database considering validly published and effectively described type species. Whereas 16S rRNA gene sequences of other non-type species were obtained from NCBI database. Multiple alignments were performed with CLUSTALW package of MEGA X (Kumar et al., 2018), where all ambiguous positions were removed in the final dataset using pairwise deletion option. Phylogenetic reconstruction and validation were performed using neighbor-joining (NJ) algorithm (Saitou and Nei, 1987) with 1000 bootstrap re-iterations using Jukes-Cantor distance model (Jukes and Cantor, 1969). Both maximum-likelihood (ML; Felsenstein, 1981) and minimum-evolution (ME; Takahashi and Nei, 2000) methods were employed to test the robustness of the trees. The sequences were submitted to NCBI GenBank under the accession numbers: MT929273 (DF22) and MT929274 (P2).

### PE Biodegradation by Isolated Bacterial Strains

The isolated bacterial strains were checked for their plastic degradation potential. The freshly grown cells (1%) were added to 100 mL of basal medium, where 10 pieces of sterile plastic films of around 2 cm^2^ each (total 0.24 g) were used in each sample as sole carbon source. They were incubated aerobically at 30 °C in shaking condition at 150 rpm. After 45 days, the set ups were examined to check the biodegradation of the plastic films. A control which had no inoculum was incubated along with the samples.

### Morphological Analysis of PE films by Atomic Force Microscopy (AFM)

Changes on the surface of PE films due to the treatment of bacteria were visualized under Atomic Force Microscope (AFM) (Agilent Technologies Inc., United States). The polythene films were taken out from the basal medium and they were thoroughly washed with 2% sodium dodecyl sulfate to clean the surface. Thereafter, the films were dried overnight at 50 °C and the dried PE films were used for examination under AFM (Gajendiran et al., 2016). The LDPE samples were analyzed with a scan speed of 1.0 Hz and a resolution of 256 × 256 pixels.

### Surface Morphology Analysis of PE Films by Scanning Electron Microscopy (SEM)

PE films were also visualized under Scanning Electron Microscope (SEM), in order to examine the surface morphology changes due to microbial action. The PE films were treated with 2% SDS and dried at 50 °C as described above in order to remove the microbial cells and associated derbies. Finally, they were gold coated, and viewed under SEM (SEM, ZEISS and Focused Ion Beam SEM, Germany) using a Cu grid at 2500X magnification (Das and Kumar, 2015).

### Fourier Transform Infrared Spectroscopy (FTIR) of PE Films

The modification of polyethylene in terms of chemical bonds due to the action of the isolated bacterial strains was assessed by Fourier Transform Infrared Spectroscopy (Thermo Scientific IR Spectrophotometer, United States). Microbially treated and untreated polyethylene films were taken out after 45 days and washed with 2% SDS and dried at 50 °C as described earlier. Finally, control and treated PEs were analyzed under the IR spectrophotometer using transmission mode (Kowalczyk et al., 2016).

## Results

### Enrichment of PE Degrading Bacterial Cells and Indication of Degradation

**FIGURE 1:**
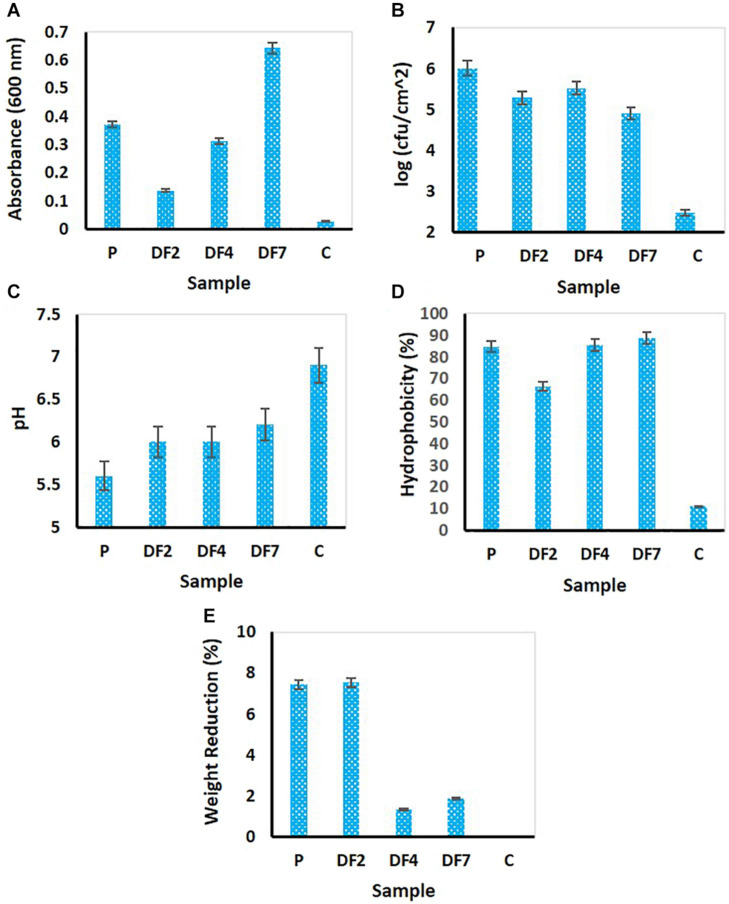
Estimation of cellular activities of microorganisms enriched with LDPE following 100 days’ incubation. **(A)** culture turbidity as measured through OD_600_; **(B)** measurement of biofilm cell growth; **(C)** change in medium pH; **(D)** measurement of cell surface hydrophobicity and **(E)** measurement of weight reduction of the LDPE beads.P: Enrichment derived from waste dumpsite; DF2, DF4, and DF7: Enrichments using drilling fluid as the source of microbes. The analyses were performed in multiple (*n* = 3–5 replicates). The mean data were used for preparation of the figure.

Enrichment of PE degrading bacterial cells was attempted by incubating LDPE beads with four different samples (namely, P, DF2, DF4, and DF7). Following 100 days’ incubation, various parameters related to cell growth and change in culture pH were measured. Cell growth was measured in terms of culture OD followed by measurement of primary indication of plastic biodegradation by monitoring weight reduction of the LDPE beads, cell surface hydrophobicity and biofilm cell growth (Figure 1).

The OD_600_ of the medium showed significant change in turbidity, thus indicating possible cell growth following 100 days’ incubation. Highest absorbance was shown by DF7, followed by *P* > DF4 and DF2 displayed the least (Figure 1A). Control set showed very low OD. Biofilm growth measured by recovering the LDPE beads from the incubation flasks showed considerable cell growth (Figure 1B). Compared to control beads with negligible CFU counts, beads from P setup yielded highest CFU of 10^6^/cm^2^, followed by the beads from DF4 culture (6 × 10^5^/cm^2^) > DF2 (3 × 10^5^/cm^2^) and DF7 (9 × 10^4^/cm^2^). It was noticed that the sample treated with landfill plastic associated soil (P) showed higher biofilm cell growth compared to the samples inoculated with drilling fluids (DF).

Compared to nearly unaltered pH of the control set, a drastic change in solution pH tending toward acidic pH from neutral pH was noted for all the biotic samples (Figure 1C). Among these, P showed a maximum decrease in pH (from pH 7.0 to pH 5.6) followed by DF2 (pH 6.0 from pH 7.0). Significant weight loss of the LDPE beads was observed in this study following their incubation (Figure 1E). Drilling fluid sample DF2 showed maximum reduction (7.54%) followed by P (7.45%), much higher than the untreated sample (no change in weight). This was a preliminary estimation of degradation since the bacteria present in the samples would utilize polyethylene, which was the only carbon source, thus leading to weight reduction of the plastic (Kyaw et al., 2012). The cell surface hydrophobicity was determined by BATH assay, which revealed that all the treated samples had more than 80% hydrophobicity except drilling fluid sample DF2 (having 66.42%) but the untreated sample (C) had only 11.11% hydrophobicity (Figure 1D). This assay showed the affinity of the bacterial cells toward the organic hydrocarbon thus highlighting the capability of enriched microbes in adhering to the polyethylene surface. This might result in enhancing the biofilm formation and degradation process (Sarker et al., 2020).

Depending on the abovementioned parameters, it was conferred that the samples P and DF2 showed better response toward biodegradation and they were selected for further analyses as well as isolation of bacterial strains. Two morphologically distinct LDPE degrading bacterial isolates were obtained as pure culture from P and DF2 enrichments. These two isolates (designated as P2 and DF22) were identified and further characterized for their LDPE degrading ability.

### 16S rRNA Gene Phylogenetic Analysis of PE Degrading Isolates

**FIGURE 2:**
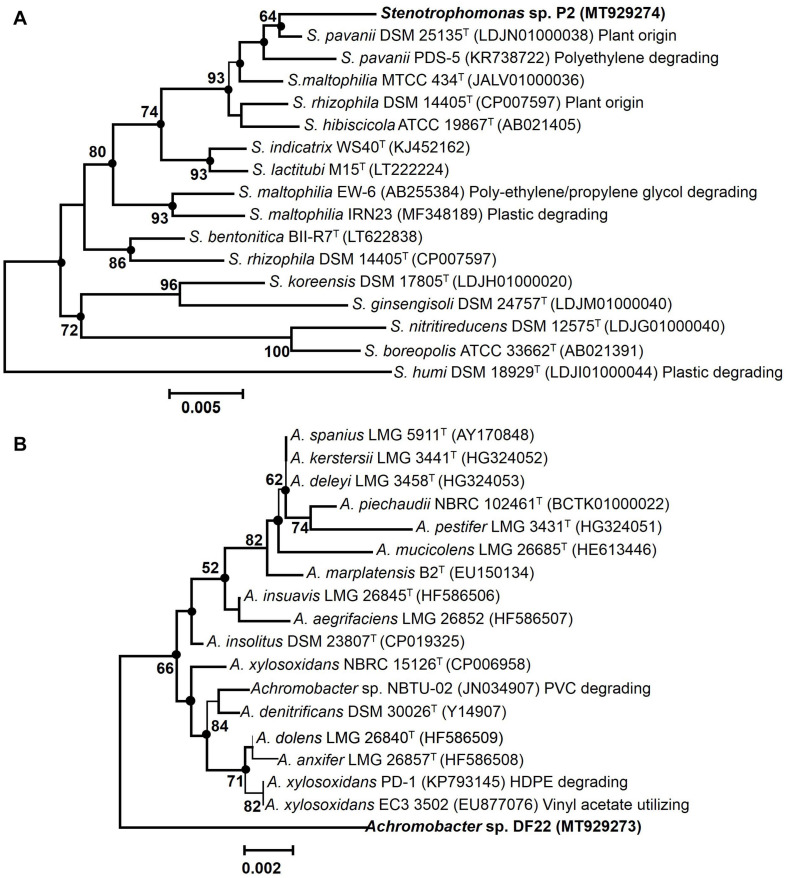
Maximum Composite Likelihood based neighbor-joining (NJ) phylogenetic reconstruction of 16S rRNA gene sequences of LDPE degrading isolates [P2 **(A)**, DF22 **(B)**, in bold] with validly described type species (T) and non-type members with various plastic degrading abilities of respective taxa. The percentage of replicate trees (>50%) based on bootstrap iterations (1000 replicates) are shown next to the branches. The filled circles indicate the corresponding branches and nodes obtained from both Maximum-Likelihood (ML) and Minimum Evolution (ME) trees. Bars, 0.005 and 0.002 represent 0.5% and 0.2% nucleotide substitutions.

EzTaxon and BLAST based comparative homology analysis of 16S rRNA gene sequences (∼1000 bp) of PE degrading isolates, P2 and DF22 showed belongingness to members of *Stenotrophomonas* and *Achromobacter*, respectively. Isolate P2 displayed maximum identities of 99.4% to type strain of *Stenotrophomonas pavanii* DSM 25135^*T*^, followed by other *Stenotrophomonas* type species (95.48–98.56%). Whereas isolate DF22 showed maximum of 97.6% identity to type strain of *Achromobacter xylosoxidans* NBRC 15126^*T*^, followed by <97.0% similarity with all other type members. Neighbor-joining phylogenetic reconstruction showed that P2 formed coherent cluster with the type strain *S. pavanii* DSM 25135^T^, while DF22 showed its maximum evolutionary closeness with several members (*A. xylosoxidans* NBRC 15126, EC3, A. insolitus DSM 23807, *etc*.) (Figure 2). Bootstrap re-sampling analysis together with combined ML and ME based phylogeny displayed similar tree topology demonstrating a strong association of *Stenotrophomonas* P2 with its closest relative *S. pavanii* DSM 25135^*T*^. Whereas, *Achromobacter* sp. DF22 was denoted to be phylogenetically distinct among *Achromobacter* spp.

Phylogenetic analysis involving various plastics (LDPE, HDPE)/polymer degrading type- and non-type members of *Stenotrophomonas* and *Achromobacter* from different habitats showed that isolate P2 was taxonomically closest to the *S. pavanii* PDS-5, which has been characterized to be a polyethylene degrading bacterium from contaminated habitat. Thus, it denoted species level affiliation of P2 to the *S. pavanii* members (Figure 2). Isolate DF22 showed distant relatedness with plastic/polymer degrading *Achromobacter* species members (Figure 2). Isolates P2 and DF22 were designated as *Stenotrophomonas* sp. P2 and *Achromobacter* sp. DF22.

### Atomic Force Microscopy and Scanning Electron Microscopy

**FIGURE 3:**
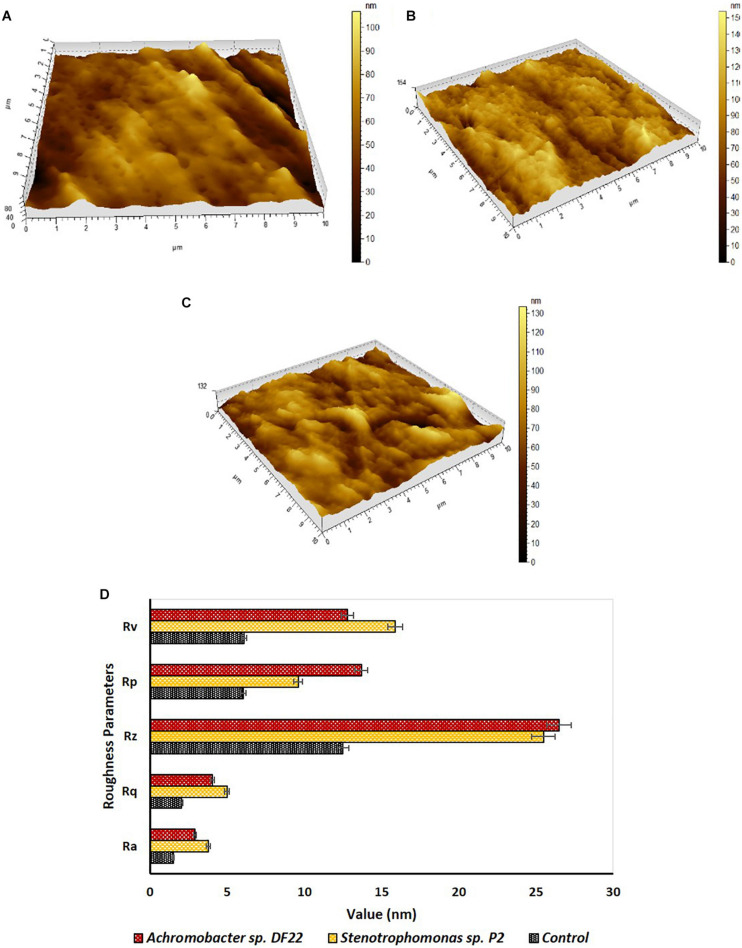
Investigation of surface topography and roughness parameters by AFM. Topography of plastic samples **(A)** control (untreated) and treated with **(B)** *Stenotrophomonas* sp. P2 and **(C)** *Achromobacter* sp DF22 after 45 days of incubation. **(D)** In comparison to Control (Black), increase in roughness parameters [average roughness (Ra), root mean square roughness (Rq), average height (Rz), maximum peak height (Rp), and maximum valley depth (Rv)] was observed for the LDPE films treated with *Stenotrophomonas* sp. P2 (orange) and *Achromobacter* sp. DF22 (brown).

Changes in surface morphology of the LDPE films were examined by Atomic Force Microscopy (AFM). The topography images of the treated films showed formation of grooves, corrosions, cracks and pits, etc., (Figures 3B,C). These changes were not observed in the untreated samples (Figure 3A). Modification in the surface morphology might be facilitated due to the action of microorganisms in the treated samples. AFM also indicated toward changes in the roughness of the surface, thus giving an overall idea on surface modification of the treated samples. The incubation for 45 days with the isolated strains caused significant increase in nano-roughness parameters, namely Ra (mean deviation of roughness), Rq (root mean square deviation of roughness), Rz (maximum height of roughness), Rv (maximum valley depth of roughness), Rp (maximum peak height of roughness) (Figure 3D), when compared to the untreated plastic. This indicated toward the formation of grooves as the result of enzymatic degradation of the LDPE (Peixoto et al., 2017). *Stenotrophomonas* sp. P2 displayed more maximum height (154 nm) than *Achromobacter* sp. DF22 (132 nm) and untreated films had the lowest (80 nm) for the same. Maximum height was the mathematical visualization of the topographs. It was noticed that *Stenotrophomonas* sp. P2 displayed more differences in maximum height, Rv, Rq, Ra and *Achromobacter* sp. DF22 had more changes in Rz, Rp (Figures 3A–D). Post incubation, roughness parameters were increased as much as two to three fold due to the action of both the strains.

**FIGURE 4:**
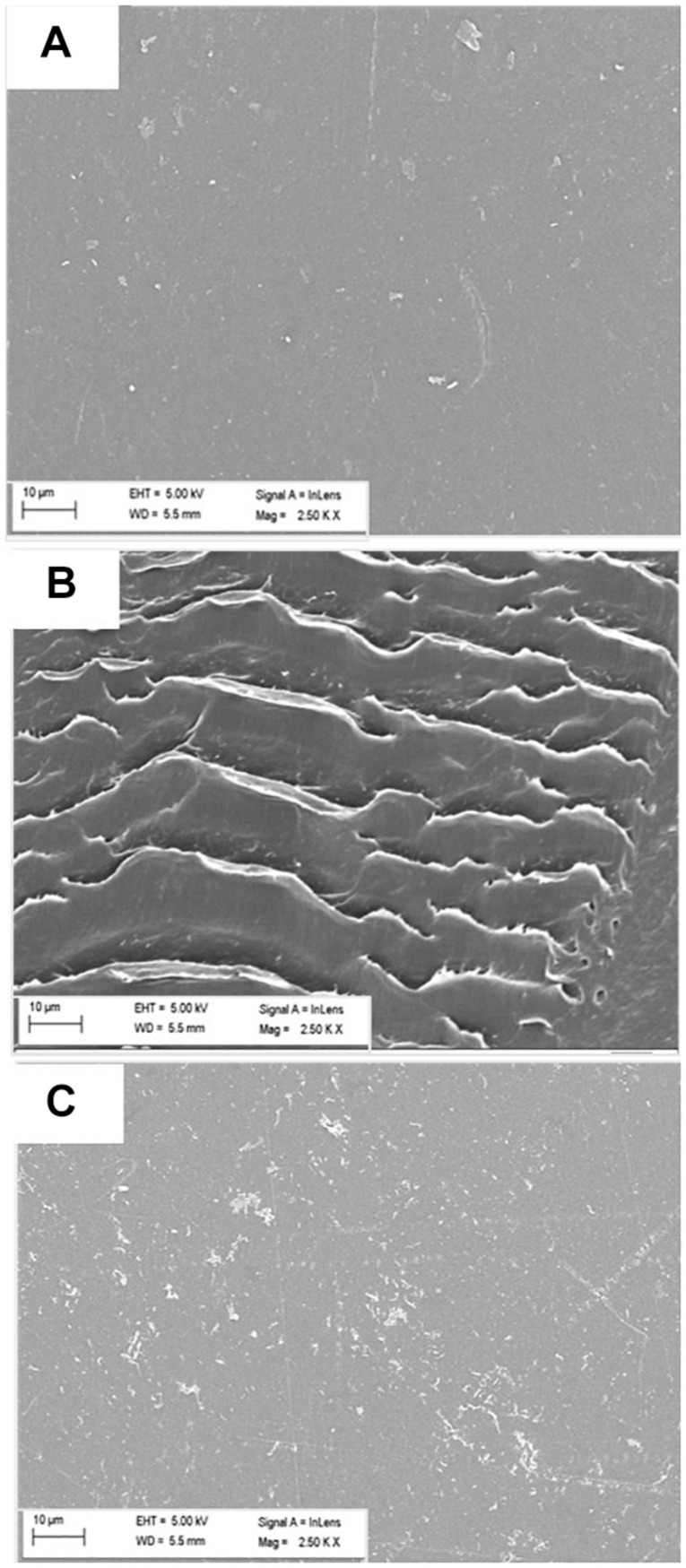
Modification of surface morphology of LDPE films **(A)** untreated, and treated with **(B)** *Stenotrophomonas* sp. P2 and **(C)** *Achromobacter* sp. DF22 observed under SEM at 2500 × amplification. Changes on the surface in terms of corrosion, crack formation, etc., were visible only for the treated samples after the incubation period.

Scanning Electron Microscopy (SEM) was performed next to further validate the LDPE surface modification due to the enzymatic action of microbes. Significant alterations on the surface of treated (with microorganisms) films were clearly observed, indicating toward the primary release of debris due to microbial action. Presence of cracks, erosions and grooves was significantly noted in the treated samples (Figures 4B,C); whereas the surface of the control or the untreated LDPE was mostly smooth (Figure 4A).

### Assay of Biodegradation by Spectroscopy

**FIGURE 5:**
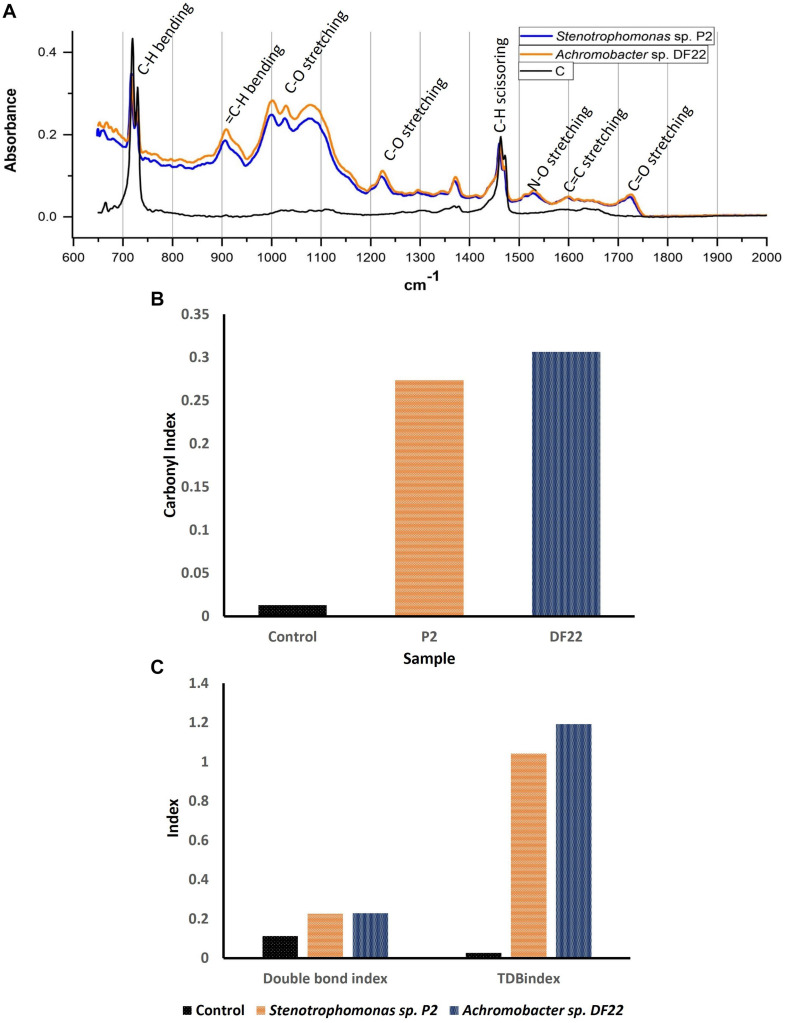
**(A)** FTIR spectra of plastic films after treating with *Stenotrophomonas* sp. P2 (Blue) and *Achromobacter* sp. DF22 (Orange) showing the clear differences in the chemical bonds compared to control (Black) due to the bacterial treatment. **(B)** Change in Carbonyl Index (CI) for LDPE treated with *Stenotrophomonas* sp. P2 (P2: Orange) and *Achromobacter* sp. DF22 (DF22: Deep blue). **(C)** Changes in Double Bond Index and Terminal Double Bond Index (TDBindex) for the LDPE films incubated with *Stenotrophomonas* sp. P2 (Orange) and *Achromobacter* sp. DF22 (Deep blue) compared to Control (Black).

The FTIR spectroscopic analysis of the LDPE films after 45 days of incubation gave several insights into the process of chemical bond modification. When compared with the untreated plastic film, changes in FTIR spectra in few unique wavelengths in the treated LDPE films were found which confirmed the modification of bonds and generation of new bonds or functional groups in the polyethylene backbone due to the biotic treatment. Majorly altered and the significant characteristic bands were found between 600 cm^–1^ and 2000 cm^–1^. Treated and untreated samples had few common peaks that belonged to polyethylene. Peaks at 720 cm^–1^ and near 1450 cm^–1^ were formed due to the long chain of C-H (bending vibration) (Yang et al., 2014). The peaks that were different from the control represented the main changes in chemical bonds and incorporation of functional groups. The formation of alkoxy groups, C-O (1000 cm^–1^ to 1090 cm^–1^); nitro groups, N-O (1500 cm^–1^ to 1600 cm^–1^); acyl groups, C-O (peak at 1220 cm^–1^) and carbonyl groups, C = O (peak near 1720 cm^–1^) was mainly found. In addition, some other modifications were observed, i.e., chain scission, H_2_C = C- (905 cm^–1^) and N-O stretching (peak at 1360 cm^–1^) (Figure 5A). The bacterial enzymes could easily utilize these groups as their function site. Also, the double bond of carbon would be more accessible by bacteria compared to C-C. Chemical bond formation and modification obtained through FTIR spectroscopy helped us to understand the mechanism of enzymatic degradation of LDPE. Apart from the graph, some indices like Carbonyl Index (CI), Double Bond Index (DBI) and Terminal Double Bond Index (TDBI) were calculated to evaluate functional bond formation and LDPE chain modifications such as oxidation, vinylene formation and chain scissioning (Figures 5B,C). Treated LDPE showed significant increase in CI as much as 15–20 fold (Figure 5B) along with 1.5–2 times increase in DBI and 30–40 fold rise in TBDI (Figure 5C). The significant increase of the indices in case of the treated samples was quite promising as a result of biodegradation. The bacteria used LDPE as their source of carbon and made the necessary changes incorporating the functional groups to modify them into simpler products like alcohol, ketone, fatty acids, etc., (Peixoto et al., 2017).

## Discussion

Samples from municipal waste dumpsite soil and bentonite based drilling fluid from a deep subsurface drilling operation were collected and used to enrich indigenous microorganisms capable of LDPE biodegradation. Microbial populations inhabiting the landfills have been already explored by several researchers for their polyethylene degrading potential (Gajendiran et al., 2016; Kowalczyk et al., 2016; Muhonja et al., 2018; Park and Kim, 2019). Regarding the microorganisms of drilling fluids used in deep subsurface drilling operations, various studies have reported their taxonomic versatility and biodegradation potential (Alekhina et al., 2007; Ozyurek and Bilkay, 2017, 2018; Bose et al., 2020). Plastic metabolizing ability of such bacteria, however, has not been explored in detail.

All the test samples were incubated aerobically in a carbon free basal medium with LDPE beads as sole carbon source for 100 days. Visible growth and turbidity in the medium post incubation confirmed the successful enrichment of LDPE degrading microorganisms. Decrease of medium pH to an acidic range suggested the metabolic activity of the enriched cells (Duddu et al., 2015). The fragmentation and depolymerization of LDPE would lead to the formation of smaller molecular weight products such as fatty acids, organic acids, etc., which might cause the decrease in pH (Das and Kumar, 2015; Karamanlioglu et al., 2017). Decrease in medium pH and increase in absorbance of the medium for all the samples established that the enriched microorganisms could utilize LDPE as sole carbon source. This increase in absorbance could also reflect the possibility of the presence of fragmented LDPEs as a result of microbial action (Chatterjee et al., 2010). These enrichment cultures and the LDPE beads were subjected to series of tests for further evaluation of their LDPE degradation potential. High biofilm cell growth and effective colonization on the surface of the treated LDPE beads in all the test samples indicated toward the enrichment of LDPE degrading microorganisms. Biofilm formation on the surface of the hydrophobic and high molecular weight plastic polymers, such as LDPE is considered as one of the most important steps of microbial degradation of these polymers (Sivan, 2011). Furthermore, it has been already reported that the cells in biofilms are known to secrete exopolysaccharides that aid in their attachment to the surface of hydrophobic plastic polymers (Wilkes and Aristilde, 2017 and reference therein). Biofilm formation enabled the microorganisms to attach and efficiently utilize the non-soluble substrate *via* various enzymatic reactions (Orr et al., 2004; Peixoto et al., 2017). High cell surface hydrophobicity in all the test samples further confirmed the biofilm formation, as the hydrophobicity of any organism has a direct correlation with its potential to bind to non-polar hydrocarbons such as polyethylene (Sanin et al., 2003; Tribedi and Sil, 2013; Sarker et al., 2020). Highest weight loss of treated LDPE beads recovered from P and DF2 samples indicated that microbial consortia enriched from these two samples were more efficient in LDPE degradation. This weight reduction must have taken place because of the enzymatic action of the enriched microorganisms attached to the LDPE surface (Kyaw et al., 2012; Gajendiran et al., 2016). These two microbial consortia also displayed quite high metabolic activity. Overall, microbial consortia enriched from both landfill and drilling fluid samples showed promising capability of LDPE degradation. Microbial consortia enriched from P and DF2 samples were found to be more efficient toward LDPE degradation. They were further used for isolation of LDPE degrading bacteria.

Two LDPE degrading bacterial isolates, P2 and DF22 were obtained as pure culture from these enrichments. The isolated strains P2 and DF22 showed closest affiliation with microorganisms belonging to *Stenotrophomonas* and *Achromobacter* respectively. Phylogenetic analysis of the isolates denoted species level affiliation of *Stenotrophomonas* sp. P2 to the *S. pavanii* members. Based on the published criteria of < 98.65% (cut-off) of 16S rDNA similarity for differentiating two species members and description of new species (Kim et al., 2014), *Achromobacter* sp. DF22 might represent a new (novel) species within the genus *Achromobacter*. S. *pavani* PDS-5 (taxonomically closest to *Stenotrophomonas* sp. P2) has been already identified as a potent polyethylene degrader (Mehmood et al., 2016). Earlier studies have also reported the ability of other microbial members (Table 1) belonging to this genus toward plastic degradation (Abe et al., 2010; Peixoto et al., 2017; Montazer et al., 2018). Besides, *Stenotrophomonas* spp. have also been used for the bioremediation of organic pollutants like fenvalerate, 3-phenoxybenzoic acid, dibenzothiophene, etc., (Chen et al., 2011; Papizadeh et al., 2011). These members were found to be distantly related to *Stenotrophomonas* sp. P2. Such phylogenetic clustering could be the result of similar evolutionary changes and shared metabolic tendency as isolate P2 showed its ability to degrade/impact the LDPE in the medium and isolated as LDPE enriched culture. Microbial members belonging to the genus *Achromobacter* have also displayed their ability toward degradation of plastic based polymers. Kowalczyk et al., 2016 has successfully tested HDPE biodegradation ability of *Achromobacter xylosoxidans*, which was isolated from landfill soil. *Achromobacter* sp. NBTU-02 has shown its potential toward biodegradation of plastics (Das et al., 2012). Along with these strains, some other strains having polyethylene degrading capabilities had been isolated from various environments and their potential toward PE biodegradation was examined by determining weight reduction, cell surface hydrophobicity, chemical modification, surface morphology changes, etc., (Table 1). *Stenotrophomonas* sp. P2 and *Achromobacter* sp. DF22 were further tested for their ability toward LDPE degradation.

**TABLE 1:** Bacterial members used for biodegradation of various polyethylene (PE) materials from diverse contaminated habitats.

Organisms	Isolation source	Plastic polymer	Incubation duration (days)	Biodegradation observations	References
*Stenotrophomonas* sp. P2	Waste dump site	LDPE	100	∼8% weight reduction, biofilm, structural deformity, surface hydrophobicity, Chemical stability change	This Study
*Achromobacter* sp. DF22	Waste dump site	LDPE	100	∼8% weight reduction, biofilm, surface hydrophobicity, structural deformity, Chemical stability change	This Study
*Stenotrophomonas pavanii*	Solid waste dump site	Modified LDPE	56	Chemical alteration	Mehmood et al., 2016
*Stenotrophomonas* sp.	Soil with plastic debris	LDPE	90	Chemical stability change	Peixoto et al., 2017
*Achromobacter xylosoxidans*	Landfill soil	HDPE	150	9% weight reduction and chemical alteration	Kowalczyk et al., 2016
*Acinetobacter baumannii*	Municipal landfill	PE	30	Biomass increase	Pramila and Ramesh, 2015
*Comamonas* sp.	Soil with plastic debris	LDPE	90	Chemical stability change	Peixoto et al., 2017
*Delftia* sp.	Soil with plastic debris	LDPE	90	Chemical stability change	Peixoto et al., 2017
*Kocuria palustris* M16	Pelagic waters	PE bags	30	1% weight reduction	Harshvardhan and Jha, 2013
*Microbacterium paraoxydans*	Clinical sample	Pretreated LDPE	60	61% weight reduction	Rajandas et al., 2012
*Pseudomonas* sp.	Mangrove soil	PE	30	20.5% weight reduction	Kathiresan, 2003
*Pseudomonas aeruginosa*	Petroleum soil	Low mol. Wt. PE	80	41% weight reduction	Jeon and Kim, 2015
*Pseudomonas* sp.	Garbage soil	PE bags	180	37% weight reduction	Usha et al., 2011
*Pseudomonas citronellolis*	Municipal landfill	LDPE	4	17.8% weight reduction	Bhatia et al., 2014
*Pseudomonas putida*	Soil with plastic debris	PE	120	9–20% weight reduction	Kyaw et al., 2012
*Rhodococcus ruber*	PE waste soil	LDPE	20–28	0.8–8% weight reduction	Orr et al., 2004
*Rhodococcus rhorocuros*	Soil	PE	180	60% mineralization	Bonhomme et al., 2003
*Rhodococcus* sp.	Waste disposal site	Pretreated PE	21	33% weight reduction	Koutny et al., 2009
*Streptomyces* sp.	River delta	Pretreated PE bags	30	slight weight reduction	El-Shafei et al., 1998
*Staphylococcus arlettae*	Soil	PE	30	13.6% weight reduction	Divyalakshmi and Subhashini, 2016
*Bacillus* sp.	Waste coal	PE	225	98% weight reduction	Nowak et al., 2011
*Bacillus sphaericus*	Shallow ocean water	HDPE/LDPE	365	3.5–10%	Sudhakar et al., 2008
*Bacillus megaterium*	Soil	Pretreated PE	90	7–10% mineralization	Abrusci et al., 2011
*Bacillus amyloliquefaciens*	Solid waste dump site	LDPE	60	11–16% weight reduction	Das and Kumar, 2015
*Bacillus subtilis* H1584	Pelagic waters	PE	30	1.5–1.75% weight reduction	Harshvardhan and Jha, 2013
*Chryseobacterium gleum*	Activated sludge	UV irradiated LPDPE	30	Chemical alteration	Jeon and Kim, 2014

**FIGURE 6:**
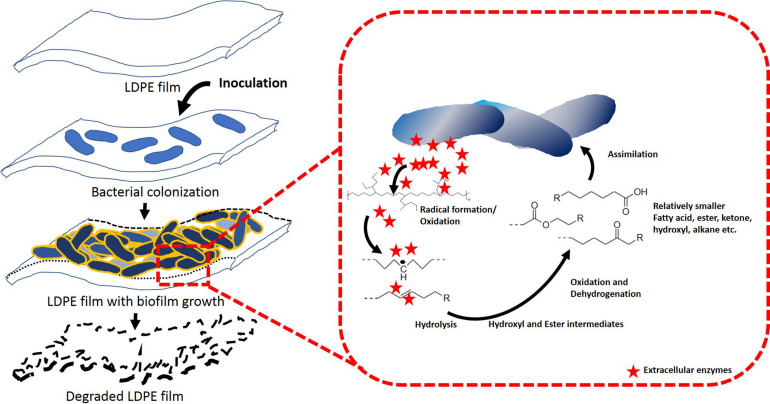
Proposed LDPE degradation mechanism by *Stenotrophomonas* sp. P2 and *Achromobacter* sp. DF22.

Both the strains were incubated aerobically for 45 days in a carbon free basal medium, using LDPE films as sole C source. Following incubation, the films were examined for the evidences of changes in surface morphology and chemical modifications by AFM, SEM and FTIR. Both, AFM and SEM analyses confirmed significant modifications on the surface of LDPE films and weakening of their physical integrity due to microbial action by both the strains (Li et al., 2020). Apart from the visible modifications, almost 2–3 fold increase in nano-roughness parameters namely Ra, Rv, Rp, Rq, and Rz added stronger evidence to the alteration of roughness of the films because of microbial action. Higher values of Rz, which is the maximum height, suggested toward the presence of big grooves and pits (Peixoto et al., 2017). All the changes also indicated toward the penetration of the bacterial cells into the LDPE film surface (Esmaeili et al., 2013). These results indicated that the LDPE degradation had been initiated by the plastic degrading strains due to their enzymatic action, where laccase, esterase, mono-oxygenase, peroxidases might play significant role (Ahmed et al., 2018; Ru et al., 2020). In order to support these results, chemical modifications of the LDPE films were determined by FTIR. Chemical modification reflected the change in carbon backbone and helped to understand the formation of functional groups on LDPE or any molecule formed during the degradation process. FTIR spectroscopy is widely used as an efficient analytical technique to identify organic, polymeric and inorganic material and their chemical conformation. This technique had been utilized in earlier studies to display the chemical changes during plastic biodegradation (Peixoto et al., 2017; Montazer et al., 2018; Li et al., 2020). New functional groups (such as alkoxy, acyl, carbonyl, and nitro) were found and modifications in terms of chain scissioning, nitro stretching and double bond formation took place. Thus, the hydrophobicity of inert LDPE was reduced due to the incorporation of functional bonds to facilitate the microbial action. Although the exact enzymatic reactions are yet to be understood, the overall process of biodegradation and enzymatic mechanism could be conceptualized from the FTIR analysis (Figure 6). Together from the increased indices (CI, DBI, TDBI) and the spectral analysis it could be inferred that the degradation process might have been initiated by the formation of radicals that helped in enhancing oxidation with the help of oxygen and water. These oxidation products were then transformed to the functional groups and vinylene group probably by Norrish type I and Norrish type II mechanisms or stabilization. Thus, the hydrophobicity of the plastic surface was reduced so that the subsequent bacterial enzymes could enhance the degradation process. Presence of alkoxy and acyl groups in the treated samples indicated ester hydrolysis and alcohol formation due to the action of microbial hydrolytic enzymes such as cutinase, lipase, esterase and alkane monooxygenase. Formation of carbonyl bonds could have occurred due to the action of laccase. These ester, alcohol, etc., could be subsequently converted to smaller fatty acids through an intermediate step of aldehyde production by dehydrogenase enzymes. Thus, smaller molecular products were formed and could be taken up by the bacteria for metabolism, leading to mineralization via β-oxidation (Ogihara, 1963; Albertsson et al., 1987; Orr et al., 2004; Szép et al., 2004; Chatterjee et al., 2010; Peixoto et al., 2017; Ru et al., 2020). These processes resulted in the breaking down of LDPE films causing the surface changes on the films.

## Conclusion

This study displayed the LDPE biodegradation potential of microbial consortium enriched (using LDPE as sole carbon source) from dumpsite and drilling fluids and the bacterial strains isolated from these enrichments. Significant microbial growth, high cell surface hydrophobicity of the enriched consortia and considerable weight reduction of treated LDPEs gave a primary indication of the biodegradation capacity of the enriched consortia. Two LDPE degrading bacterial strains affiliated to *Stenotrophomonas* sp. and *Achromobacter* sp. were isolated as pure culture from P and DF2 enrichments. SEM and AFM analyses confirmed that both the strains were successful in altering the cell surface morphology of the LDPE beads. The overview of possible mechanism of LDPE biodegradation by these two strains was also established in this study. FTIR analysis suggested that series of chemical changes starting from oxidation followed by dehydrogenation led to the breaking down of LDPE into smaller molecules, which could be subsequently utilized by these two strains for their metabolism. Complete microbial composition of the enrichments can be analyzed through next generation sequencing based approach and would help us to identify the complex network of biological systems involved in LDPE degradation. This would enable us to develop a stable microbial consortium capable of LDPE degradation, which could be utilized for large-scale biodegradation of plastic. Further investigation on the metabolic pathways, enzymatic reactions and metabolites would help us understanding the exact mechanism of biodegradation by these bacterial strains to develop *in situ* process for LDPE biodegradation.

## Data Availability Statement

The datasets presented in this study can be found in online repositories. The names of the repository/repositories and accession number(s) can be found in the article/supplementary material.

## Author Contributions

AD performed the experiments, analyzed the data, and drafted the manuscript. HB helped with the phylogenetic analysis and manuscript preparation. BM helped in analytical methods and data analysis. PS conceived the idea, arranged funds, supervised all the experiments, data analysis, and manuscript writing. All authors contributed to the article and approved the submitted version.

## Conflict of Interest

The authors declare that the research was conducted in the absence of any commercial or financial relationships that could be construed as a potential conflict of interest.

## Footnotes

1 https://www.ezbiocloud.net/identify
